# Inequity Aversion Negatively Affects Tolerance and Contact-Seeking Behaviours towards Partner and Experimenter

**DOI:** 10.1371/journal.pone.0153799

**Published:** 2016-04-15

**Authors:** Désirée Brucks, Jennifer L. Essler, Sarah Marshall-Pescini, Friederike Range

**Affiliations:** Comparative Cognition, Messerli Research Institute, University of Veterinary Medicine, Vienna, Medical University of Vienna, University of Vienna, Vienna, Austria; Utrecht University, NETHERLANDS

## Abstract

Inequity aversion has been proposed to act as a limiting factor for cooperation, thus preventing subjects from disadvantageous cooperative interactions. While a recent study revealed that also dogs show some sensitivity to inequity, the underlying mechanisms of this behaviour are still unclear. The aim of the current study was threefold: 1) to replicate the study by Range et al. (2009, *PNAS*, **106**, 340–345); 2) to investigate the emotional mechanisms involved in the inequity response by measuring the heart rate and 3) to explore the link between inequity aversion and cooperation in terms of behaviours shown towards the partner dog and towards the experimenter who caused the inequity. Dog tested in dyads were alternately asked to give their paw and were either equally or unequally rewarded by the experimenter. After each social test condition, we conducted food tolerance tests and free interaction tests in which the subjects’ social behaviour towards the partner and the experimenter were observed. As in the previous study, subjects refused to continue giving their paw when only the partner was rewarded, but not when both dogs were rewarded with rewards of different quality. Although subjects did not react to this quality inequity during the test, we did find reduced durations of food sharing in the subsequent tolerance test, indicating that dogs perceived the inequity but were not able to react to it in the test context. Moreover, subjects avoided their partner and the experimenter more during the free interaction time following unequal compared to equal treatment. Despite the clear behavioural reactions to inequity, we could not detect any changes in heart rate. Results suggest that inequity aversion might in fact be mediated by simple emotional mechanisms: sharing a negative experience, like inequity, might reduce future cooperation by decreasing the likelihood of proximity being maintained between partners.

## Introduction

Inequity aversion–the negative response to unequal outcomes, has now been studied for more than a decade in non-human animals (for a review see [1]). Inequity aversion is thought to play a role in cooperation, in that it is advantageous to keep track of effort and subsequent payoff of cooperative interactions in order to withdraw from a cooperative partnership if there is no balance in the long-term. In this respect, inequity aversion is thought to be a stabilizing factor in regard to cooperation [2]. Despite the potential importance of inequity aversion for cooperation, contrasting results have been found when testing non-human animals, with some studies finding positive evidence for inequity aversion (e.g. [3–6]), while others finding no such support (e.g. [7–10]). Some of the contradictory findings can likely be explained by differences in the experimental approaches (for a critical review see [11]), but explanation for some of the other contrasting results remain elusive (e.g. [10,12]. Further studies, both testing a wider variety of species, and refining the experimental paradigms could help to address these discrepancies.

Canids are potentially interesting in order to extend our understanding of the evolution of inequity aversion since they show high degrees of cooperation among group members (e.g. wolves [13,14] and African wild dogs [15]; for a review on group hunting in carnivore species see [16]). Indeed, recent research revealed that domestic dogs show at least a simple form of inequity aversion ([17], but see [8]). In this study, pairs of dogs from the same household were asked to give their paw alternately to an experimenter and, in some conditions, were unequally rewarded for performing this action. The dogs reacted aversely when their companion was rewarded whilst they were not, whereas they were quite willing to continue to give the paw to the experimenter if they were tested alone without the partner being present and likewise if both–subject and partner–were not rewarded. These results therefore ruled out that dogs’ behaviour was purely guided by the frustration of not receiving a reward. Rather it indicated that dogs showed a sensitivity to inequity. However, the dogs were indifferent to the differential distribution in terms of the quality of the reward as well as the differential effort requested by the researcher, in that they continued performing the task when their companion received a more preferred reward or they had to work for the reward whilst their companion was given a ‘present’. This absence of a reaction seems to separate dogs from several other species, which do show a negative reaction to differences in reward quality and invested effort (reaction to different reward quality: chimpanzees [18], bonobos [12], capuchin monkeys [19], cottontop tamarins [20], long-tailed macaques [21], rhesus macaques [5], crows and ravens [6]; reaction to different effort required: capuchin monkeys [19], long-tailed macaques [21], crows and ravens [6]).

Overall, while we have some evidence that dogs react to unequal treatment at least in one paradigm, we lack knowledge regarding the underlying mechanisms of this behaviour. For example, is the dogs’ reaction to unequal treatment a precursor form of a more complete inequity sensitivity (including ‘quality’ reward inequity) or do other factors (e.g. the will to please the experimenter and/or lack of inhibitory control) override dogs’ reaction to these other forms of inequity during execution of the specific task? Dogs’ willingness to follow human commands might have ‘forced’ them to continue giving their paw independent of the resulting reward as long as there was one. Alternatively, dogs’ inhibitory control might have been insufficient to allow them to interrupt the behaviour of giving the paw since in the quality inequity condition they still received a reward for giving the paw (although the non-preferred reward).

Moreover, a direct link between inequity aversion and cooperation has been hypothesized [22] and has gained support from studies utilising cooperation paradigms to test for inequity aversion. Capuchin monkeys as well as cottontop tamarins have been shown to engage in a cooperative problem-solving task only as long as the outcome was equal or at least equally shared between the two individuals [20,23]. Chimpanzees on the contrary, seem to avoid inequity by negotiating over unequal outcomes before enganging in cooperation thus preventing the break-down of cooperation [24]. Apart from these studies, we still have no evidence of the interplay between inequity aversion and cooperation in the more often used exchange tasks. Since inequity is caused by the experimenter and cannot be influenced by the test partners, the situation in exchange tasks is very different from cooperation paradigms in which subjects can decide beforehand whether to cooperate with the partner or not for unequal outcomes. If we assume that inequity aversion has been selected for stabilizing cooperation, following unequal situations, we would expect to find a negative effect also on other behaviours relating to cooperation, for example, tolerance in a food context, or affiliation towards the experimenter and/or partner. In the current study we aimed to address these questions from three perspectives.

Firstly, we aimed to *replicate the overall pattern of results and extend previous findings* by Range and colleagues [17], by making a series of modifications to assess whether dogs would also show a sensitivity to ‘quality inequity’ (i.e. negative reaction to receiving a less preferred reward than the partner) and if not, what may be the reason for it. We modified the procedure by (*i*) conducting individualized food preference tests prior to testing to ensure that the value of the two rewards used was clearly different for each subject. In fact, since no preference tests had been conducted in Range et al.’s study (who relied on owners’ reports of food preference), dogs’ inequity response may have been hindered by a lack of preference between the presented alternatives: sausage vs. bread. (*ii*) We removed warm-up trials (i.e. handing out one high quality reward to each dog prior to testing) at the beginning of test sessions, since the inclusion of these has been criticised as potentially enhancing the subject’s expectation of a reward during testing, thus leading to extinction of the paw-giving behaviour instead of eliciting inequity aversion [8]. And (*iii*) in contrast to experiment 1 of the previous study, where the partner received the low value reward both in the equity (subject received the same reward) as well as reward inequity condition (subject received no reward), here we rewarded the partner with a high quality reward to test whether we could induce a stronger inequity effect by making the rewarding scheme even more unequal. Furthermore, instead of the effort control condition from Range et al. [17], we tested dogs in a food control condition, more commonly used in primate studies (e.g. [4]). In the food control condition, the experimenter pretends to give the dogs the more preferred reward, but instead exchanges it again for the less preferred reward, which is then handed to the dog. This food control condition was included since it directly induces frustration by violating the reward expectancies. Thus allowing us on the one hand to further test whether the obtained responses could be explained by frustration rather than inequity aversion and on the other hand to assess whether dogs can potentially react to the differences in reward quality in the test context as it would be necessary for an reaction to the quality inequity condition. If dogs’ lack of quality inequity is due to the inability to perceive the quality differences in the test context, we would expect to continue giving paw also in this individual contrast condition. However, if dogs are not able to socially compare outcomes of different quality, we hypothesize that they would not refuse in the quality inequity condition but would do so in the individual contrast condition, since this condition only requires a comparison between reward qualities at the individual level (e.g. as in [25]).

Second, we looked at the *emotional mechanisms* involved in dogs’ reaction to inequity. As we know from humans, unequal treatment elicits strong negative emotions, like anger and spite (e.g. [26,27]). This negative reaction is also mirrored in certain physiological measures–decreased heart rate as well as increased cortisol levels have been shown to be associated with humans’ rejections of unequal offers during cooperation games [28]. In order to understand whether similar emotions are involved in non-human animals’ responses to inequity, additionally to the dogs’ behavioural response, we also measured the subject’s heart rate during the inequity test. If dogs’ reaction to inequity is to some extent comparable to humans’, we would expect that also dogs will show a lower heart rate in the inequity conditions compared to the baseline condition.

Third, to better understand how dogs perceive the inequity paradigm and to investigate the *link between inequity aversion and other cooperative behaviours*, we tested the tolerance of subjects towards their partner and towards the experimenter after the different test conditions. If dogs’ behaviour in the inequity test is linked to other cooperative behaviours (i.e. food sharing), we would expect a reduced tendency to cooperate following unequal treatment. Moreover, most experimental paradigms for studying inequity aversion (i.e. token exchange [3]) involve not only two individuals performing a joint interaction but a third party which is responsible for handing out rewards. Consequently, it is not clear whether the subject views the partner or the experimenter delivering the reward as being responsible for the unequal outcome [18, 23]. No study so far has addressed this issue. Therefore, we investigated the social behaviours towards the partner dog and towards the experimenter during an unrestricted interaction time. We hypothesized, that if dogs consider the experimenter as being responsible for the outcome they should show no changes in tolerance towards their partner in the above mentioned tolerance tests but show avoidance behaviour towards the experimenter after unequal test conditions. On the contrary, if dogs perceive the task as a joint interaction between themselves and their partner, and view the partner as being responsible for the outcome, we would expect a reduced tolerance towards their partner after unequal conditions and no/minor changes in behaviour towards the experimenter.

## Methods

### Subjects

Twenty dog dyads (mean age + SE: 5.3 + 2.7 yrs.) of various breeds, including mixed breed dogs were tested in this study (see Table 1 for individual characteristics), however, 3 dyads did not complete testing and were excluded (reasons for exclusion of dyads: 1 dog showed no food preference, 1 owner stopped participation, 1 dog showed no more motivation to complete the task after two test sessions). Only dogs living in the same household for at least 1 year were tested. Precondition for participation in the study was that dogs would give their paw on command 15 times to the experimenter with and without being rewarded for it (5x rewarding, 5x no reward, 5x rewarding). This order of rewarded and non-rewarded trials was deliberately chosen in order to avoid dogs from getting frustrated about not receiving a reward on their very first encounter with the experimenter and test situation. Only dogs showing no sign of food aggression were included in the study. As further exclusion criterion for analyses dogs had to complete at least 20 trials in the assessment condition (working alone for the low-value reward) to ensure sufficient motivation. Two dogs had to be excluded due to lack of motivation (i.e. completed less than 20 trials in the assessment condition). All tests were conducted between January and October 2014 at the Clever Dog Lab, in a test room (7 x 6 m) and always by the same experimenter. Ethical approval was obtained from the ‘Ethik und Tierschutzkomission’ of the University of Veterinary Medicine Vienna (Protocol Number: 08/08/97/2013) and owners were required to sign a consent form prior to testing.

**Table 1:** Individual characteristics of dogs that completed testing (*N* = 34) and low value rewards (LVR) used in each dyad.

Dyad	Name	Sex	Breed	Age (yrs.)	LVR
1	Achuk	F	Chesapeake Bay Retriever	8.3	dry food
1	Elrond	M	Chesapeake Bay Retriever	4.6	dry food
2	Aiko	M	Australian Shepherd	2.4	dry food
2	Emely	F	Bernese Mountain Dog	1.7	dry food
3	Baja	F	Australian Shepherd	1.1	carrots
3	Daimony	F	Australian Shepherd	6.7	carrots
4	Bessy	F	Border Collie	2.6	dry food
4	Eve	F	Border Collie	6.0	dry food
5	Bella	F	Bernese Mountain Dog	6.5	cheese
5	Brandy	F	Bernese Mountain Dog	3.0	cheese
6	Chasie	F	Border Collie	4.8	carrots
6	Gatsby	M	Border Collie	3.5	carrots
7	Cole	M	Border Collie	6.4	dry food
7	Esprit	F	Border Collie	3.7	dry food
8	Cookie	F	English Cocker Spaniel	6.9	dry food
8	Dino*	M	Cuvac—Mix	2.5	dry food
9	Emily	F	Border Collie	5.7	carrots
9	Ziva	F	Border Collie	2.1	carrots
10	Flappi	F	Pumi-Mix	4.6	carrots
10	Joey	M	Pointer-Mix	6.1	carrots
11	Geischa	F	Australian Shepherd	8.1	carrots
11	Yuuki	M	Australian Shepherd	2.1	carrots
12	Luke	M	Border Collie	8.0	cornflakes
12	Quismo	M	Border Collie	6.1	cornflakes
13	Luna	F	Siberian Husky	1.2	cheese
13	Talie	M	Siberian Husky	2.8	cheese
14	Mago	M	Golden Retriever	9.1	carrots
14	Tika	F	Husky—Mix	6.1	carrots
15	Nessie	F	Terrier—Mix	13.6	dry food
15	Flamme*	M	Berger des Pyrenées	6.4	dry food
16	Pippilotta	F	Irish Terrier	7.1	dry food
16	Poquita	F	Galgo Español	4.1	dry food
17	Sokrates	M	Bardino–Mix	7.9	dry food
17	Ultimo	M	Border Collie	4.2	dry food

* dogs did not show sufficient motivation and were only considered in their partner role (i.e. equity and food control condition excluded from whole dyad)

### Food Preference Test

Prior to testing a food preference test was conducted with every dog. First, the owner was asked which food their dogs really like (high value reward = HVR; sausage for all dogs) and which food their dogs do not particularly like but will still eat and work for (low value reward = LVR; see Table 1). Owners were only limited in their reward type suggestions in terms of feasibility (e.g. no fresh meat or human dishes). The same food rewards were used for both dogs within a dyad. Second, we conducted individual preference tests to verify the owner’s observations. The owner was instructed to sit on a chair and to keep the dog on a leash in front of him/her. The experimenter visibly baited two differently coloured plastic lids (black and white) with the two food types within a 1.20m distance from the dog. Then she leaned towards the dog, letting it sniff both lids before leaning back again and putting them on the ground equidistant to the dog. The lids were placed with 60–70 cm distance from the dog and 50 cm from each other. As soon as the experimenter removed her hands from the lids and looked down, the owner released the dog by dropping the leash. Only one choice was allowed and as soon as the dog touched one lid, the other lid was covered and removed by the experimenter while the dog ate the reward. 12 trials were conducted per session, alternating the side of the reward to prevent dogs from developing a side bias. The starting position of the HVR (i.e. left or right) was counterbalanced across dogs. If the dog chose the HVR in 9 trials (binomial: *p* < 0.02), it was considered a clear preference. In case no preference was found within 3 sessions, other food types were chosen and new preference tests conducted on another test day.

### Inequity Test

**Fig 1:**
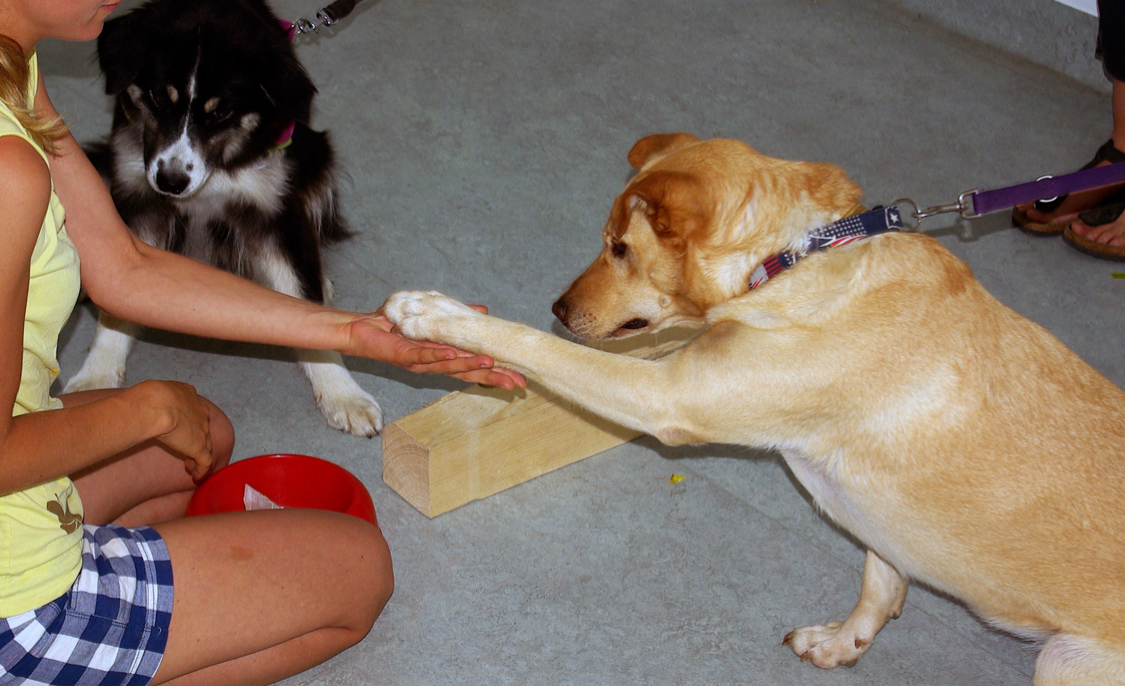
Setup of the paw task. The red bowl in front of the experimenter contained both reward types. The owner was standing behind the dogs.

Following the procedure of Range et al. [17], the two dogs were seated next to each other, facing the experimenter while the owner was standing passively behind the dogs. Both dogs were kept on leashes of the same length and were separated by a wooden block (60 x 10 x 10 cm) lying on the ground. The experimenter was kneeling at a distance of 50 cm in front of the dogs with a bowl (30 cm diameter) containing sufficient pieces of both food types. The reward types in the bowl were separated by a piece of cardboard, always having the LVR in the front and HVR in the back partition. The bowl was positioned between the legs, clearly visible to the dogs (see Fig 1).

In contrast to Range et al., no HVR was given to the dogs prior to testing, instead, the task started immediately when the dogs were seated and the experimenter sat down in front of them. Apart of this difference, the procedure was identical to the previous study: Both dogs were asked alternately to give their paw (verbally and by holding a hand out), always starting with the partner dog. The experimenter avoided eye contact with the dogs during testing. If a dog successfully gave its paw, the experimenter took a reward from the bowl, held it up in front of the dogs for each to clearly see (< 20 cm from the noses of the animals) and then handed it over to the actor, before asking the other dog for its paw. The dog could either receive a piece of LVR, a piece of HVR or no food at all for completing the action depending on the test condition (see Table 2). If the actor (partner or subject) did not give its paw after two seconds of holding the hand outstretched, the command was repeated. The paw-command was repeated for a maximum of 10 times with calling the dog’s name once after the 5^th^ repetition. If the actor still refused to give the paw, the test session was terminated. Dogs were only asked for their paw if they were in a sitting position, if they were lying or standing, they were asked to sit first (verbally and index finger outstretched). The sit-command was repeated 10 times with calling the dog’s name once after the 5^th^ repetition. If the dog still refused to sit after 10 repetitions, the dog was ignored for 5 trials, in which the partner dog was asked to give the paw 5 times (these trials were not considered in the analyses). After these trials, the dog was addressed again and asked to sit for 10 times (same procedure as before). If the dog still did not obey after 10 repetitions, the test was terminated. This procedure was generally only applied to the subject dogs since the partner dogs complied immediately with the commands. All dogs were tested by the same experimenter who kept her behaviour constant across dogs and conditions.

**Table 2:** Test conditions for paw task.

**Condition**	**Subject**	**Partner**
*Social Conditions*		
Equity (ET)	LVR	LVR
Quality Inequity (QI)	LVR	HVR
Reward Inequity (RI)	No reward	HVR^+^
Food Control (FC)	HVR moved, LVR given	HVR moved, LVR given
*Asocial Conditions*		
Assessment Control (AC)	LVR	--- *
No Reward Control (NR)^§^	No reward	--- *

* In order to control for the movement of the food in the asocial condition, a piece of LVR was picked up from the bowl and moved to the empty partner’s side as if the partner were being rewarded, then it was moved back again and placed back into the bowl before the subject’s trial started (see Range et al., 2009).

^+^ In experiment 1 of Range et al (2009), the partner received the LVR in this condition

^§^ In experiment 2 of Range et al (2009), the subject was tested with a partner that also received no reward.

### Test Conditions

Dogs were tested in six different conditions differing in the reward scheme between subject and partner dog (see Table 2). We conducted 60 trials per session, alternating between subject and partner dog (i.e. 30 trials per dog). Compared to experiment 1 of Range et al. [17], we incorporated an additional test condition, the food control (FC). In this condition, the experimenter picked up a piece of HVR after the paw was given and lifted it to the dogs’ head height but then put it back in the bowl and handed the dog only a LVR. The same procedure was done for the partner dog, resulting in the same outcome for both dogs (both LVR) as in the equity condition. The order of test conditions was semi-randomized with the exception that the first session was never started with neither the reward inequity (RI) nor the no reward control (NR) condition to avoid complete frustration. Each dog served as partner and as subject during the test; however, the roles were only reversed after a dog was tested in all conditions (i.e. in the last test session). Furthermore, the asocial control conditions were always tested in one block (i.e. within one session) starting with the assessment condition (AC) and ending with the no-reward control condition (NR). A second experimenter, sitting 2 m away from the dogs, on the side of the room, directly noted each completed trial on a data sheet; furthermore, all test sessions were video recorded for behavioural coding and analyses. Two conditions were tested on one day with a 10-minute break in-between conditions. Immediately, after each social test condition, a food tolerance test was conducted (description see below), whereas after the non-social conditions, the break time started directly after finishing the test. Following the tolerance tests, the dogs were allowed to move freely around the room, with both the owner and experimenter present (see below for details of the break time). If a dog stopped giving the paw in the test, the paw-command was reinforced again with HVR for 5 times at the very end of the appointment after the test was finished (i.e. after the 2^nd^ break time). Dogs were tested one to three times a week with at least one day in-between tests.

### Heart Rate Measurements

Prior to each test session, a digital heart rate monitor (Polar® RS800CX) consisting of a transmitter attached to an elastic belt and a watch-like receiver device was attached to each dog. The polar device has been shown to reliably measure dogs’ heart rate [29,30]. The belt was attached to the dogs’ chests. However, to facilitate recording, the dogs’ fur was first made wet with water and then ultrasound transmission gel was applied on the skin and the electrode to promote conductivity. The belt was tightly, but still comfortably, strapped around the dog’s chest, positioning the electrode on the left side close to the dog’s heart. After ensuring that both dogs’ belts were working, we started the data collection and synchronized the polar-watch with the video recording. Dogs were habituated to wearing the belt on a prior occasion (for 10 min) before the first test session. Indeed, all dogs showed normal behaviours while wearing the device from the first time it was applied, therefore no further training with the belt was needed. After testing, the heart rate data was transmitted from the watch to the computer software (Polar® ProTrainer 5) for further analyses.

### Tolerance Test

**Fig 2:**
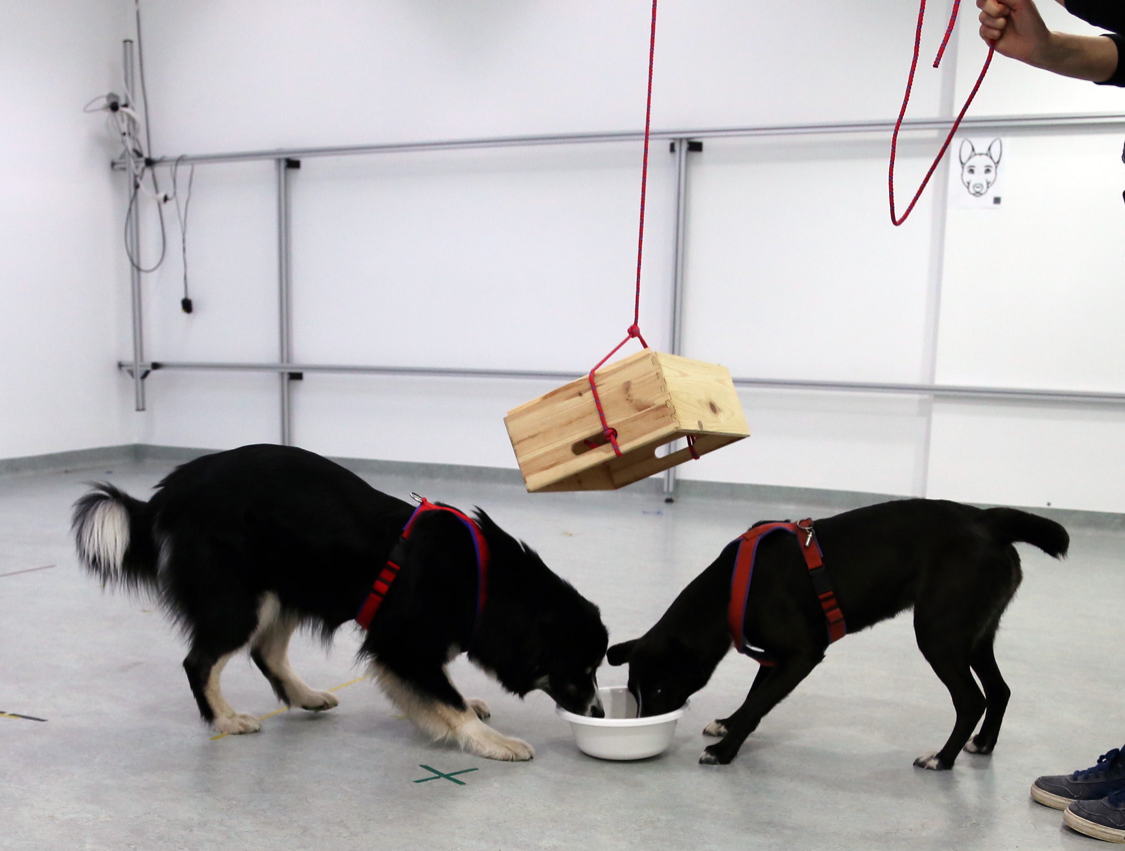
Setup for tolerance test. Dogs waiting for the box to be pulled up in order to access to the bowl containing pieces of sausages. The experimenter lifted the box when both dogs were within a 10cm radius of it.

Directly following each social test condition (within 1 min after the last trial), a food tolerance test was conducted. This test consisted of both dogs feeding from a single bowl (20 cm diameter) filled with 1.5 sausages cut into 2 cm slices. The bowl was covered with a wooden box (see Fig 2) to ensure that the dogs would reach the bowl at the same time, it was only lifted if both dogs were within a 10 cm radius of the box.

In one habituation trial at the beginning of the study, the dogs were familiarized with the movement of the box. 10 dogs were afraid of the box movements and therefore the test was conducted without using a box for these dyads. In these cases, the owner held both dogs by the collar whilst the experimenter placed food on the floor equidistant to both dogs. Dogs were then released simultaneously on command from the experimenter. The tolerance test ended when both dogs left the bowl (i.e. more than one body length away).

### Experimenter and Partner- Directed Behaviours Following Inequity Test

After the tolerance test, the experimenter removed all food items from the test room (always leaving and re-entering the test room through the same door) and sat down on the floor with the owner. The dogs were off leash from the previous tolerance test and not restricted or handled in any way. The starting point for this interaction test was set to the moment when owner and experimenter touched the floor with their knees. Owner and experimenter sat opposite one another, 2 meters apart and remained in this position for the subsequent 10 minutes. The owner was instructed to behave passively (i.e. not giving verbal or gestural commands) and also the experimenter behaved passively towards the dogs. The dogs were allowed to move freely around the room if they approached one of the persons they were petted, however, they were free to leave that person again at any time.

### Data Coding and Analyses

All tests were video recorded and then coded using the Solomon coder beta (2015 by András Péter; http://solomoncoder.com/). We coded the following variables for the paw task: number of times the paw was given, number of paw prompts, duration of gazing to the partner’s side (i.e. turning head to partner) and number of stress behaviours (i.e. yawning, licking, scratching) exhibited. Since test sessions differed in length based on the number of paws that were given, we corrected the variables ‘gaze to partner’ and ‘stress signals’ by calculating the average of each parameter by the number of paws given within the respective test session. Videos of 5 test sessions were missing due to technical problems. For these sessions we used the data that were noted directly during testing (i.e. number of times the paw was given and paw prompts) but no further behavioural data was available. Furthermore, one dog’s QI condition needed to be excluded because the partner stopped giving the paw during the test.

In case of the heart rate measurements, we analysed the mean heart rate (HR) in beats-per-minute (bpm) since of the available parameters this seems to be the most reliable when using the polar device [29,31]. To exclude extreme artefacts in the data, we corrected the HR data manually by confining a minimum and maximum threshold. As dogs’ natural HR range seems to be within 30–240 bpm (e.g. [32]), we deleted single measures that were not within this range. Since several measurement errors occurred during testing, in particular because of the dogs’ changing body postures, we did not analyse the heart rate variability, which is very sensitive to missing or wrong values (e.g. [31]). Two dogs needed to be excluded from HR analyses as they suffered from heart disease.

For the tolerance test we coded the latency to approach the box/ bowl (i.e. time from releasing dogs until in 10 cm radius to box or bowl), the duration of co-feeding (i.e. both dogs feeding from the bowl simultaneously without showing aggressive behaviours) and feeding alone (i.e. only one dog feeding) from the bowl, as well as the frequency of submissive (i.e. lips licking, low body posture, turning away from partner) and dominant behaviours (i.e. growling, snapping, standing stiff) during the tolerance test. Moreover, during the interaction time we coded the first 5 minutes in terms of the dogs’ behaviours to their partner and the experimenter. We coded the latency to initiate contact with the experimenter (either standing close and looking at her face (< 1m) or direct body contact) and with the partner dog (i.e. within one body-length of the partner). Furthermore, we looked at the proportion of time spent in close proximity (within one body length) of the experimenter and partner dog. The break was carried out after each tolerance test; however, only the break following the NR, ET and RI condition was analysed to test our hypotheses. Unfortunately, for 12 dogs one or two break time recordings (total of 17 sessions) were missing.

Statistical analyses were conducted in R 3.1.2 [33] using the package ‘lme4 (1.1–7)’ [34] for linear mixed models. Our response variables directly obtained from the inequity test (i.e. number of paws and paw-prompts) showed a strong positive skew, due to dogs mostly completing 30 trials in the different conditions. Because this data was not randomly distributed across conditions but systematically biased by the different reward schemes and inconsistent number of paw prompts in each condition, we used non-parametric statistics with correction for multiple testing to analyse the response variables. To investigate the influence of conditions on continuous behavioural factors, we ran linear mixed models (LMM) and used stepwise backward regression analyses to retain only significant effects in the model using likelihood ratio tests (LRT). We ran all LMMs with the fixed factors: condition (factor (ET, FC, QI, RI, AC, NR) and session (continuous) to control for a potential session effect. As response terms we used: average duration of gaze to partner per trial (log-transformed), mean heart rate, latency to approach experimenter (log-transformed) and partner dog (log-transformed), proportion of time spent in proximity to experimenter (log-transformed) and partner (arcsine-square root transformed). If normal distribution could not be achieved using data transformation, non-parametric statistics were used. In this case we corrected for multiple testing using the sequential Bonferroni correction method. If p-values did not remain significant after correction it is indicated in the result section. 20% of the videos (paw task, tolerance test and break time) were coded by a second person (Intra-class correlation coefficient (ICC, consistency): paw task: number of paws given: ICC = 0.99, number of commands: ICC = 1.00, stress signals: ICC = 0.76, gaze: ICC = 0.84; tolerance test: co-feeding: ICC = 0.92, feed alone: ICC = 0.93; break time: latency to initiate contact with experimenter: ICC = 0.89, latency to initiate contact with partner dog: ICC = 0.88, duration close to experimenter: ICC = 0.90, duration close to partner: ICC = 0.91).

## Results

### Refusals Across Conditions

**Fig 3:**
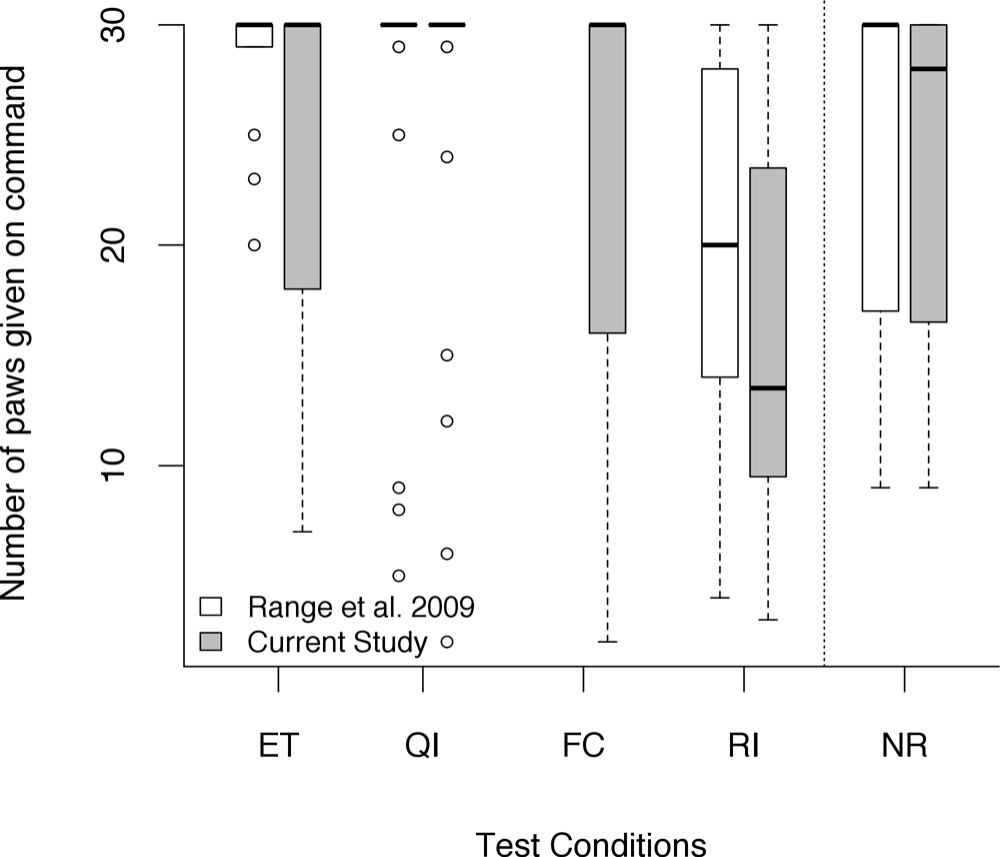
Number of times the paw was given on command per test condition in Range et al. [17] (*N* = 29) and the current study (*N* = 32). Circles show outliers, black bars indicate median values, whiskers display upper and lower hinge, and boxes show the interquartile range.

The number of ‘giving the paw’ differed between conditions (Friedman *χ*^*2*^ = 49.62, *df* = 5, *N* = 30, *p* < 0.001; Fig 3). Dogs refused to give paw more often in the RI condition compared to the ET condition (Wilcoxon signed-ranks test: *T* = 20, *N* = 30, *p* < 0.001). This refusal rate was more pronounced if the partner received the reward (RI) compared to nobody receiving it (NR) (Wilcoxon signed-ranks test: *T* = 43, *N* = 32, *p* < 0.001). We found neither a difference in the number of times the paw was given in the FC (Wilcoxon signed-ranks test: *T* = 35, *N* = 30, *p* = 0.284) nor in the QI condition (Wilcoxon signed-ranks test: *T* = 49, *N* = 31, *p* = 0.456) compared to the baseline ET condition. Session order (e.g. 1, 2, 3 or 4^th^ session) did not affect the number of paws given in each condition (Kruskal-Wallis Test: ET: *df* = 2, *p* = 0.228; FC: *df* = 2, *p* = 0.159, QI: *df* = 3, *p* = 0.617, NR (only 1, 2, 3^rd^ session): *df* = 2, *p* = 0.369 and RI (only 2, 3, 4^th^ session): *df* = 3, *p* = 0.482). Likewise, we did not find an effect of whether the dogs started the test as partner or subject dog (Mann- Whitney *U*-test: *U = 513*.*5*, *N1* = 32, *N2* = 31, *p* = 0.805). Additionally, we found no effect of the reward received in the previous test session (HVR or LVR) on the number of paws given (Wilcoxon Test: *T* = 1451.5, *N* = 32, *p* = 0.149).

A similar pattern could be observed when looking at the average number of paw prompts across conditions (Friedman: *χ*^*2*^ = 49.22, *df* = 5, *N* = 30, *p* < 0.001). Dogs had to be asked to give paw more often in the RI condition compared to the baseline ET condition (Wilcoxon signed-ranks test: *T* = 14, *N* = 30, *p* < 0.001). However, no difference emerged between the other social conditions (Wilcoxon signed-ranks tests: FC-ET: *T* = 78, *N* = 30, *p* = 0.962; QI-ET: *T* = 69, *N* = 31, *p* = 0.314). Additionally, more paw-prompts were necessary in the RI compared to the NR condition (Wilcoxon signed-ranks test: *T* = 63.5, *N* = 32, *p* = 0.014).

### Comparison with Range et al. [17]

When directly comparing the dogs’ performance in the current study with the study by Range et al. [17], we found only a significant difference in refusals in the RI condition (Mann- Whitney *U*-test: *U = 142*.*5*, *N1* = 32, *N2* = 29, *p* = 0.037): dogs stopped giving their paw earlier in the current study compared to Range et al.’s study (Fig 3). No differences emerged when comparing the other conditions between studies (Mann- Whitney *U*- test: ET: *U = 384*.*5*, *N1* = 30, *N2* = 29, *p* = 0.361; QI: *U = 439*.*5*, *N1* = 30, *N2* = 29, *p* = 0.835; NR: *U = 412*.*5*, *N1* = 32, *N2* = 29, *p* = 0.432).

### Behavioural Variables

We found a difference in stress behaviours across conditions (Friedman: *χ*^*2*^ = 16.11, *df* = 5, *N* = 30, *p* = 0.007). Dogs showed more stress behaviours in the RI condition compared to the ET condition (Wilcoxon signed-ranks test: *T* = 89, *N* = 30, *p* = 0.027, n.s. after Bonferroni correction). The occurrence of stress behaviours in the RI condition was also significantly higher than in the NR condition (Wilcoxon signed-ranks test: *T* = 70, *N* = 32, *p* = 0.008). No differences emerged when comparing the other conditions to the baseline (Wilcoxon signed-ranks test: QI-ET: *T* = 118, *N* = 30, *p* = 0.368; FC-ET: *T* = 215, *N* = 30, *p* = 0.162).

**Fig 4:**
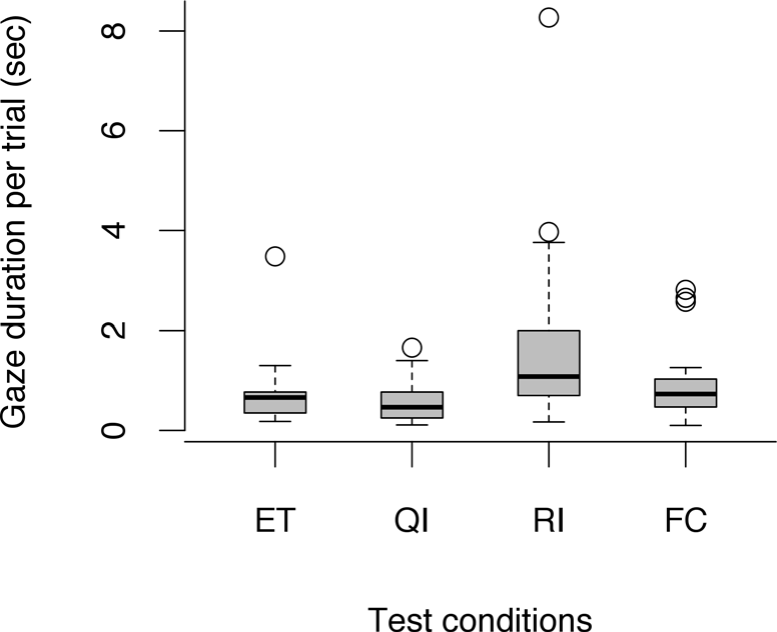
Average gaze duration to partner per trial across social test conditions. Circles show outliers, black bars indicate median values, whiskers display upper and lower hinge, and boxes show the interquartile range.

Furthermore, the average gaze duration per trial was affected by test conditions (LRT: *χ*^*2*^ (5) = 65.32, *p* < 0.001; Fig 4) but also by session number (LRT: *χ*^*2*^ (1) = 4.30, *p* = 0.038), however, there was no interaction between these two factors (LRT: *χ*^*2*^ (5) = 6.17, *p* = 0.290). Dogs showed longer gaze durations at the partner in the RI condition compared to the ET condition (LMM: *β* = 0.59, *SE* = 0.15, *df* = 145, *t* = 3.89, *p* < 0.001). No difference emerged in gaze durations between the other conditions and the baseline (LMM: QI-ET: *β* = -0.30, *SE* = 0.15, *df* = 146, *t* = -1.94, *p* = 0.054; FC-ET: *β* = 0.21, *SE* = 0.14, *df* = 144, *t* = 1.45, *p* = 0.151). Moreover, dogs showed longer gaze durations during later test sessions (LMM: *β* = 0.09, *SE* = 0.05, *df* = 154, *t* = 2.05, *p* = 0.042).

### Heart Rate

Test Conditions as well as session number did not affect the mean HR of dogs (LRT: test conditions: *χ*^*2*^ = 3.77, *df* = 5, *p* = 0.583; session number: *χ*^*2*^ = 0.34, *df* = 1, *p* = 0.560). Following this, we decided to take a closer look at the HR of those dogs that showed a clear reaction in the RI condition (i.e. more than 5 trials difference between RI–NR condition, *N* = 23). However, also in this subgroup no difference emerged in HR between conditions (LRT: *χ*^*2*^ = 3.34, *df* = 5, *p* = 0.647).

### Tolerance Test

**Fig 5:**
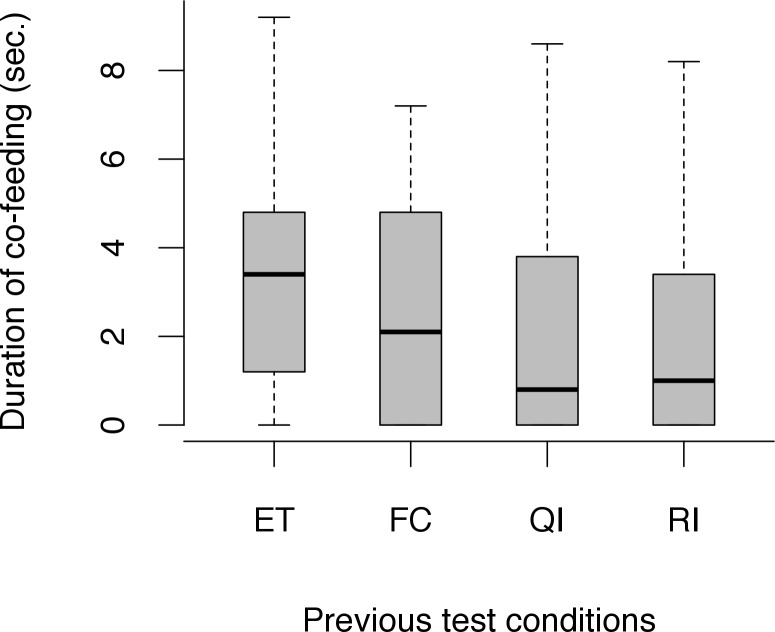
Duration of co-feeding following each social test condition (*N* = 30). Black bars indicate median values, whiskers display upper and lower hinge, and boxes show the interquartile range.

The duration of co-feeding differed in relation to the previously tested condition (Friedman: *χ*^*2*^ = 104.05, *df* = 23, *N* = 30, *p* < 0.001; Fig 5). Dogs showed less co-feeding following the RI condition in comparison to the ET condition (Wilcoxon signed-ranks test: ET-RI: *T* = 268, *N* = 30, *p* < 0.001). Interestingly, dogs also showed less co-feeding following the QI condition when comparing it to the tolerance test following the baseline ET condition (Wilcoxon signed-ranks test: *T* = 238 *N* = 29, *p* < 0.001). The amount of co-feeding did not differ between tolerance tests carried out after the FC and ET conditions (Wilcoxon signed-ranks test: ET-FC: *T* = 198.5, *N* = 30, *p* = 0.068). A negative correlation between the number of times the paw was given in the inequity test during the RI condition and the duration of subsequent co-feeding emerged (Spearman rank correlation: *r*_*s*_ = -0.45, *N* = 32, *p* = 0.012), suggesting that dogs were even less tolerant if they experienced inequity for a longer time period. As most of the dogs worked for 30 trials in the QI condition, only a tendency emerged between number of times the paw was given in the test and the duration of subsequent co-feeding (Spearman rank correlation: *r*_*s*_ = -0.37, *N* = 31, *p* = 0.051).

Furthermore, the duration of feeding alone from the bowl differed between conditions (Friedman: *χ*^*2*^ = 72.55, *df* = 23, *N* = 30, *p* < 0.001). The subject dogs that were not rewarded during the RI condition fed alone from the bowl more often compared to after the equity condition (Wilcoxon signed-ranks test: ET-RI: *T* = 23, *N* = 30, *p* = 0.012). However, the subjects receiving the lower reward in the quality inequity condition did not show an increased duration of feeding alone afterwards (Wilcoxon signed-ranks test ET-QI: *T* = 31, *N* = 30, *p* = 0.328). The latency to approach the box or bowl did not differ between conditions (Friedman: *χ*^*2*^ = 4.81, *df* = 3, *N* = 30, *p* = 0.186). Since aggressive and submissive behaviours were only rarely observed during the tolerance test (mean frequency: aggressive behaviours = 0.039, submissive behaviours = 0.010) we were not able to analyse those variables.

### Experimenter and Partner- Directed Behaviours Following Inequity Test

**Fig 6:**
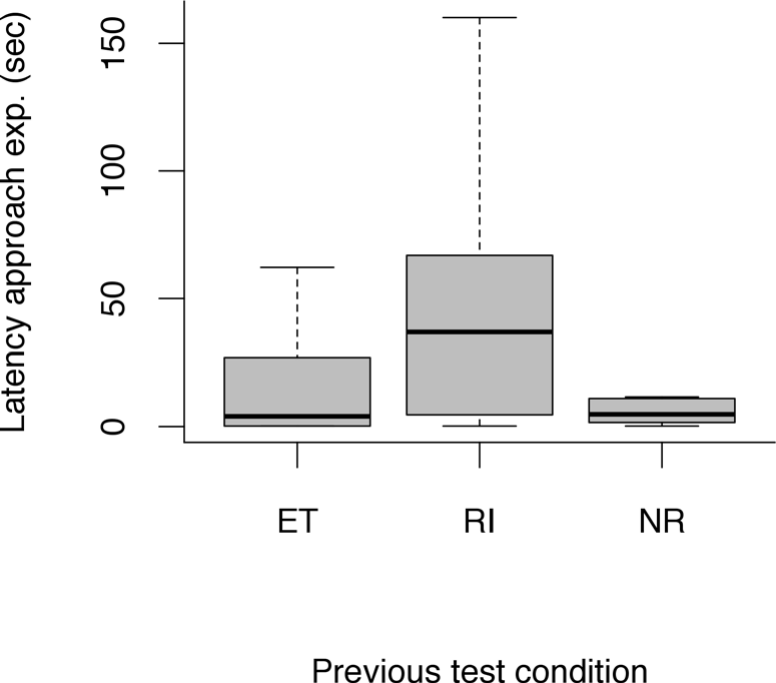
Latency to approach the experimenter in the break time following the equity (ET), reward inequity (RI) and no-reward control (NR) condition. Black bars indicate median values, whiskers display upper and lower hinge, and boxes show the interquartile range.

The subject’s latency to approach the experimenter differed between conditions (LRT: *χ*^*2*^ = 7.68, *df* = 2, *p* = 0.02). During the break following the RI condition (and the respective tolerance test), it took the subjects longer to approach the experimenter (LMM: *β* = 1.57, *SE* = 0.58, *df* = 49, *t* = 2.72, *p* = 0.009) than in the break after the ET condition. However, there was no difference between the ET and NR condition in the latency to approach the experimenter during the break (LMM: *β* = 0.50, *SE* = 0.60, *df* = 48, *t* = 0.83, *p* = 0.412) (see Fig 6). Moreover, the latency to approach the partner dog did not differ between conditions (LRT: *χ*^*2*^ = 0.80, *df* = 1, *p* = 0.061). Furthermore, session number did not affect the approach latency (LRT: experimenter: *χ*^*2*^ = 2.03, *df* = 1, *p* = 0.154; partner dog: *χ*^*2*^ = 0.80, *df* = 1, *p* = 0.371).

**Fig 7:**
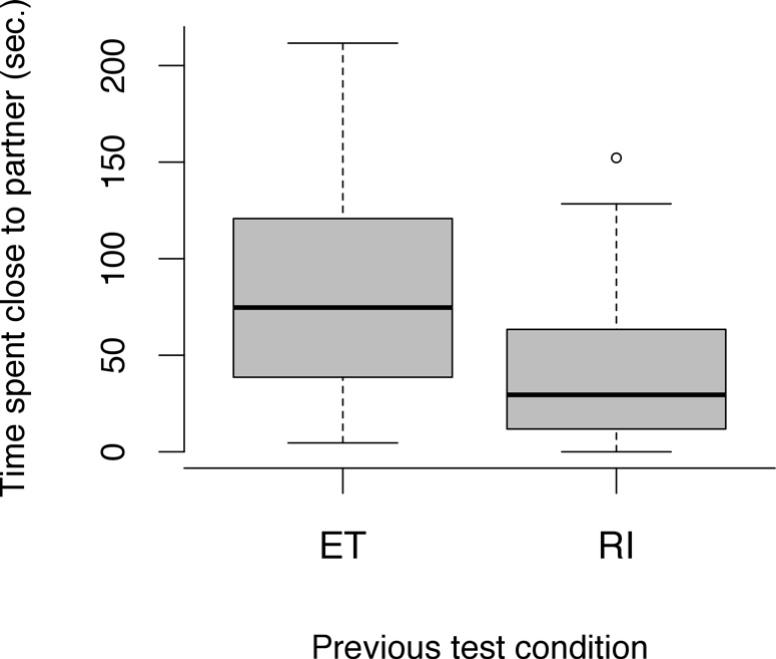
Proportion of time spent in close proximity to the partner dog during the break time following the equity (ET) and reward inequity (RI) condition. Circles show outliers, black bars indicate median values, whiskers display upper and lower hinge, and boxes show the interquartile range.

Moreover, the proportion of time spent in close proximity to the partner dog was affected by the previous test condition (LRT: *χ*^*2*^ = 7.41, *df* = 1, *p* = 0.006) but not by the session number (LRT: *χ*^*2*^ = 0.02, *df* = 1, *p* = 0.900). Dogs spent less time in close proximity to their partner in the break following the RI condition compared to the break after the ET condition (LMM: *β* = -0.34, *SE* = 0.12, *df* = 24, *t* = -2.97, *p* = 0.007) (see Fig 7). The proportion of time spent in proximity to the experimenter, however, did not differ between conditions (LRT: *χ*^*2*^ = 0.76, *df* = 2, *p* = 0.684) and was not affected by session number (LRT: *χ*^*2*^ > 0.01, *df* = 1, *p* = 0.981).

## Discussion

Our goals in the current study, were to 1) replicate some of the findings by Range et al. [17] and 2) to extend these finding by including an additional control and a follow-up test to further understand longer-term effects of the unequal reward distribution. We found similar to the orginal study that dogs were inequity averse if they saw that their partner got a reward whilst they themselves did not, but that they continued to work if the reward distribution differed in quality rather than quantity. This result is in line with the fact that dogs also did not show more refusals if the individual reward expectations were not met as in the food control condition. Interestingly, however unequal treatment based on food quality did alter dogs’ behaviour after the test situation in the tolerance test. Finally, while the behavioural differences observed between test conditions were reflected in the dogs’ exhibition of stress behaviours, we could not detect these apparent emotional changes across conditions in variation of their mean heart rate.

While some studies suggest that mere frustration about not getting a preferred reward can explain behaviours supposedly reflecting inequity aversion (e.g. [9,10]), our results indicate that this is not the case in dogs for multiple reasons. Firstly, if frustration was the primary factor driving the dogs refusual to give the paw, we would not have expected a difference between the reward inequity and the asocial no-reward condition. However, dogs refused to give the paw earlier, needed more paw-commands, showed more stress signals and gazed more to their partner when they saw that the partner received a reward. These results are in line with the previous study [17] where the same overall difference emerged both in a comparable set-up as here as well as in a setup where dogs were tested in the reward inequity condition and a social control condition in which the partner was present but both animals did not receive a reward. Second, dogs did not show a reaction to the food control condition, which explicitly induced frustration in both dogs. Third, our additional behavioural measures following the inequity test show that dogs avoided the experimenter for a longer time, if the experimenter rewarded the partner dog compared to the asocial condition, in which the rewarded partner was absent, suggesting that mere frustration for not getting a reward did not drive dogs’ behaviour towards the experimenter. Additionally, the reduced duration of co-feeding following both inequity conditions further indicates that dogs reacted to inequity on a social level–namely towards their social partner—and not merely to a violation of their reward expectations, which would be shown only on an individual level and consequently should not affect the behaviour towards the partner (e.g. as in [9,10]).

When directly comparing results from our current study with Range et al.’s study, we found that dogs refused to give their paw even earlier in the reward inequity condition in the current study compared to the previous one. Additionally, in contrast to Range et al., we found a difference in gaze duration between conditions, as dogs looked longer at their partner in the reward inequity condition than during the baseline. Inducing more inequity by handing the high value, instead of the low value reward to the partner, could have caused an even greater violation of expectation, explaining the earlier refusal and the increased gaze duration by the subject. However, the latter finding concerning the increased gaze duration in the reward inequity condition have to be treated with caution, since Range et al. did not find a difference in gaze duration between their conditions and thus our finding might be caused by subject dogs being more interested in the movement of the high value reward. Nonetheless, these results show that dogs’ reaction to reward inequity can be additionally manipulated by the reward quality given to the partner dog.

Although we carefully considered individual food preferences, no reaction emerged in the quality inequity condition, which is consistent with the previous study. While dogs were clearly capable of discriminating between the two reward types in the food preference test and showed a reduced duration of co-feeding behaviour towards the better rewarded partner, their lack of responding to the quality inequity needs to be based on other factors. While it has been shown that dogs are able to exhibit distinct negative reactions when individual reward expectations are violated [25], this was not the case in our study, and this in spite of the fact that the difference in reward quality was even greater for some of our dogs (carrot vs. sausage) compared to those in Bentosela et al.’s study [25] (dry food vs. pieces of liver). Consequently, we propose two main factors that may be responsible for the lack of a reaction to reward quality manipulations in these two conditions: 1) dogs were unable to discriminate between the different food qualities delivered to the self and the partner in the test context (e.g. experimenter hands concealed the food quality) and/or 2) dogs lack the inhibitory control ability which would allow them to refrain from an action (giving the paw) which delivers a reward (regardless of its quality). Further studies manipulating the food visibility on the partner’s side and investigating the interplay between inhibition abilities and inequity aversion, are needed, to disentangle these alternative explanations.

While at the behavioural level dogs clearly reacted to unequal treatment, we did not find a corresponding change in heart rate measures. However, these negative results have to be seen with caution for several reasons. First, although the Polar® system has been validated for the use in dogs, at least in stationary positions or during exercise on a tread mill [29], we faced some difficulties with obtaining high quality recordings when dogs were sitting or lying, which happened often during the inequity test. Second, several studies have shown that the polar device is not always a reliable tool for measuring dogs’ heart rate and the resulting data needs correction (e.g. [31]). Accordingly, we decided beforehand to only focus on the least error-prone heart rate variable, namely the mean heart rate across sessions, however, it could be worthwhile in future studies to look at other heart rate parameters, such as the heart rate variability, which has been shown to also change during unequal test situations in humans [28]. This is the first study that implemented heart rate measures during an inequity test in non-human animals, thus we cannot compare results to other non-human animals and, due to the measurement problems, at present cannot draw final conclusions as regard physiological changes during unequal treatment in dogs.

The most interesting addition of the current study is the observation that the inequity treatment significantly affected dogs’ subsequent behaviour towards both the conspecific partner and the experimenter. Food sharing, a form of prosocial behaviour is thought to be linked to cooperation in that unequal sharing after a cooperative interaction should lead to decreased future cooperation between partners. Consequently, food sharing in terms of co-feeding should occur more often if the situation has been equal beforehand (e.g. [35]). As predicted, more co-feeding occurred if the previous test situation was an equal one (e.g. equity and food control condition) and less co-feeding was observed following unequal conditions (e.g. reward inequity and quality inequity condition). Interestingly, the duration of co-feeding was linked to the amount of experienced inequity, as dogs showed less co-feeding, when they experienced the inequity over a longer time period. Although, dogs did not show a measurable reaction to quality inequity in the test context, we did find a reduced duration of co-feeding afterwards. This indicates that dogs perceived the quality inequity but were not able to respond to it during the test situation possibly due to limits in their inhibitory control abilities. Nonetheless, the question remains who was responsible for the decreased tolerance behaviour: the subject or the partner? Following the reward inequity condition, the unrewarded dog monopolized the bowl more often compared to the equity condition, in which both dogs received the same reward. However, after the quality inequity condition neither the unequally treated subject nor the better-rewarded partner monopolized the bowl more often. Consequently, it remains elusive whether the inferiorly treated dog avoided the partner or vice versa. A possible explanation for this difference could be that the quality inequity condition was not perceived as negatively as the reward inequity condition, thus not eliciting the same strong reactions. Alternatively, partner dogs might also show a reduced motivation in accessing the rewards in the tolerance test after having received rewards during the inequity conditions. However, this explanation is unlikely because the motivation to get access to the reward–as measured in the latency to approach the bowl–did not differ between conditions. And additionally, we found no difference in the duration of co-feeding between the two inequity conditions, which would have been the case if the partner dog was less interested in the food after getting much more rewards in the quality inequity condition than in the reward inequity condition, due to the subject dog refusing to continue earlier in the latter condition. Furthermore, dogs avoided their partner dog and also the experimenter in the break time following the reward inequity more compared to the control condition suggesting that dogs’ aversion to inequity and the subsequent avoidance of the partner and the experimenter is not only confined to the test situation but also lasts for some time afterwards. A frustration based explanation for the avoidance of the experimenter can be ruled out since dogs approached the experimenter slower in the reward inequity than in the non-social condition in which likewise no rewards were handed out to the subject. These results suggest that subjects at least partially attributed the inequity situation to the partner dog and perceived the task as a joint interaction–involving both the experimenter and the partner.

While it has been proposed that inequity aversion acts as a mechanism to promote long-term cooperative relationships in a way that it allows individuals to withdraw from cooperative interactions if these yield no equal payoff [22], only two studies so far have tested this hypothesis. Brosnan et al. [23] showed that capuchin monkeys continued to cooperatively pull an unequally baited tray as long as the outcome was subsequently equally shared between partners. Cronin and Snowdon [20] tested cottontop tamarins in a similar cooperative problem-solving task and found that tamarins cooperate more if the outcome is equal. These studies show that cooperation breaks down if one individual consistently dominates the outcome, hence demonstrating how inequity aversion can stabilize cooperation. Nonetheless, these findings do not explain the very basic link between inequity aversion and cooperation. In revealing that 1) dogs’ food tolerance towards their partner seemed to decrease following unequal treatment in the inequity test and 2) this further affected the vicinity to the partner dog and the human experimenter in the subsequent break time, we can suggest how the link between inequity aversion and cooperation might work.

As Brosnan [22] already hypothesized, no higher cognitive abilities are needed for this to occur, since the negative experience with a partner (e.g. confirmed also by the higher rates of stress-related behaviours) during a joint action, is sufficient to induce avoidance and subsequently a reduced tendency to cooperate with the partner in the near future. This conclusion in turn implies that the ability to exhibit inequity aversion does not necessarily require consciously keeping track of past interactions, but rather may be mediated by the positive or negative emotions induced by such situations.

In conclusion, our results suggest that dogs show more than just a precursor form of inequity aversion, in that they are indeed sensitive to quality inequity as shown in other species. However, lack of inhibitory control and dogs’ sensitivity to human signals may override their capacity to show an overt refusal of a reward in this condition. Importantly, we could show a direct link between inequity aversion and the subsequent changes in tolerance towards the partner in terms of food sharing and proximity. Suggesting that the modulating role of inequity aversion on cooperation may be as simple as a reduced tendency to spend time with a partner with whom they have shared an emotionally negative, unequal interaction with. Similar measurements should be adopted in future studies, in order to reveal further insights into the underlying mechanisms and implications of inequity aversion in other non-human animals.

## Supporting Information

S1 File Data set for paw task and tolerance test. (XLSX)

S2 File Data set for interaction time. (XLSX)
